# Characterization of MRI White Matter Signal Abnormalities in the Pediatric Population

**DOI:** 10.3390/children10020206

**Published:** 2023-01-24

**Authors:** Katharina J. Wenger, Caroline E. Koldijk, Elke Hattingen, Luciana Porto, Wiebke Kurre

**Affiliations:** 1 Institute of Neuroradiology, University Hospital Frankfurt, Goethe University, 60528 Frankfurt am Main, Germany; 2 Gynacology Department, Buerger Hospital, 60318 Frankfurt am Main, Germany; 3 Institute of Diagnostic and Interventional Radiology/Neuroradiology, Municipal Hospital Passau, 94032 Passau, Germany

**Keywords:** white matter abnormalities, myelination, pediatric imaging, etiology, magnetic resonance imaging

## Abstract

(1) Background and Purpose: The aim of this study was to retrospectively characterize WMSAs in an unselected patient cohort at a large pediatric neuroimaging facility, in order to learn more about the spectrum of the underlying disorders encountered in everyday clinical practice. (2) Materials and Methods: Radiology reports of 5166 consecutive patients with standard brain MRI (2006–2018) were searched for predefined keywords describing WMSAs. A neuroradiology specialist enrolled patients with WMSAs following a structured approach. Imaging characteristics, etiology (autoimmune disorders, non-genetic hypoxic and ischemic insults, traumatic white matter injuries, no final diagnosis due to insufficient clinical information, “non-specific” WMSAs, infectious white matter damage, leukodystrophies, toxic white matter injuries, inborn errors of metabolism, and white matter damage caused by tumor infiltration/cancer-like disease), and age/gender distribution were evaluated. (3) Results: Overall, WMSAs were found in 3.4% of pediatric patients scanned at our and referring hospitals within the ten-year study period. The majority were found in the supratentorial region only (87%) and were non-enhancing (78% of CE-MRI). WMSAs caused by autoimmune disorders formed the largest group (23%), followed by “non-specific” WMSAs (18%), as well as non-genetic hypoxic and ischemic insults (17%). The majority were therefore acquired as opposed to inherited. Etiology-based classification of WMSAs was affected by age but not by gender. In 17% of the study population, a definite diagnosis could not be established due to insufficient clinical information (mostly external radiology consults). (4) Conclusions: An “integrated diagnosis” that combines baseline demographics, including patient age as an important factor, clinical characteristics, and additional diagnostic workup with imaging patterns can be made in the majority of cases.

## 1. Introduction

Magnetic resonance imaging (MRI) is widely used for the identification of white matter pathologies with the presence of visible white matter signal abnormalities (WMSAs). Signals from standard imaging sequences, such as T2-weighted (T2W), T1-weighted (T1W), and fluid-attenuated inversion recovery (FLAIR) can be used to evaluate their patterns [1,2,3,4,5,6]. The spectrum of underlying pathologies itself is broad [7]. Any process entailing changes in the chemical composition of myelinated axons, unmyelinated axons, and glial cells can present as WMSAs on MRI [8].

When classifying white matter disorders, the first discriminator is whether the underlying cause is acquired or heritable. Acquired forms can be divided according to the type of cause into autoimmune disorders, infectious white matter damage, toxic injuries of any kind, traumatic injuries, white matter damage caused by tumor infiltration/cancer-like disease, and non-genetic vascular insults [9]. The classification of hereditary white matter disorders is more complex and their categorization has changed over time. According to a panel of leading experts in the field (consensus publication in 2015), the term leukodystrophy should refer to heritable disorders affecting the white matter of the central nervous system which display glial cell or myelin sheath abnormalities [10]. In a follow-up publication in 2017, Van der Knaap and Bugiani proposed a classification system for leukodystrophies that depends predominantly upon the primary involvement of any white matter constituent [11]. Next to leukodystrophies, the 2015 expert panel defined categories for hereditary disorders with strong evidence for primary neuronal involvement and prominent systemic manifestations which overshadow the WMSAs (so-called “genetic leukoencephalopathies”) [10], inherited disorders with vasculopathy as a primary disorder causing WMSAs [10], and inborn errors of metabolism in which the clinical manifestations of systemic illness predominate, but brain MRI can detect significant WMSAs [10].

The aforementioned known white matter pathologies, acquired or heritable, often present with characteristic imaging patterns. However, given that the image morphology is partially overlapping, interpretation relies on the clinical context, including knowledge of baseline demographics, clinical characteristics, and additional workup. WMSAs that are not interpreted as the direct cause of the presenting symptoms, but are rather incidental findings, are often categorized as “non-specific” [12,13]. 

Over the past two decades, the rapidly expanding use of MRI in children with neurological impairments of unknown etiology has revealed a substantial number of WMSAs of known and unknown origin [14,15]. The aim of this study was to retrospectively characterize WMSAs in an unselected patient cohort at a large pediatric neuroimaging facility using standard MRI sequences. In order to learn more about the spectrum of the underlying disorders encountered in everyday clinical practice, we evaluated imaging characteristics, etiology, as well as age and gender distribution of white matter lesions.

## 2. Materials and Methods

### 2.1. Study Design and Data Preparation

This retrospective study was approved by the Institutional Review Board (IRB) of the University Hospital Frankfurt (No 85/19). Consecutive pediatric patients (age ≤ 18) who underwent brain MRI at our and referring hospitals between December 2006 and February 2018 were identified in the radiological information system (RIS) by filtering for exam type and searching for predefined keywords in the respective radiology reports. Predefined keywords were: “white matter lesion”, “white matter abnormality”, “leukodystrophy”, “leukoencephalopathy”, “demyelination”, “demyelinating encephalomyelitis”, “chronic inflammatory disease”, “inflammatory lesion”, “gliosis”, and “multiple sclerosis”.

A structured approach to imaging evaluation was taken by a neuroradiologist (W.K., >10 years of experience in pediatric neuroimaging) to confirm or dismiss imaging findings in the written radiology reports. The checklist included age at the time of neuroimaging, gender, indication for neuroimaging, WMSA pattern, WMSA location, and presence of pathological contrast enhancement on post-contrast T1W images, as well as any information arising from the patients’ medical records available in the clinical information system (CIS) and follow-up imaging. Patients were excluded from the study in cases where the imaging expert could not confirm the presence of at least one white matter lesion on MRI sequences or in cases of iatrogenic (postoperative) WMSAs. All remaining patients were categorized by their final clinical diagnosis. Categories were chosen in the context of the literature cited in the introduction section:

- Autoimmune disorders (Group 1);
- Non-genetic hypoxic and ischemic insults (Group 2);
- Traumatic white matter injuries (Group 3);
- Patients with no final diagnosis due to insufficient clinical information (Group 4; mostly patients referred by other hospitals requesting a second opinion on a prior imaging exam);
- Non-specific WMSAs (Group 5);
- Infectious white matter damage (Group 6);
- Leukodystrophies (Group 7);
- Toxic white matter injuries (Group 8);
- Inborn errors of metabolism (Group 9);
- White matter damage caused by tumor infiltration/cancer-like disease (Group 10).

There were no patients with a final diagnosis that could have been categorized as “genetic leukoencephalopathy” or “inherited disorder with vasculopathy as a primary disorder”. 

### 2.2. Magnetic Resonance Imaging

MR imaging at our hospital was performed on clinical 1.5 Tesla (T) and 3T MR scanners (Siemens Medical Solutions, Erlangen, Germany). Scanner types and protocols were subject to change over time. Imaging at referring hospitals was performed on numerous scanner types with varying MR protocols. However, all MRI protocols included a group of basic MRI sequences available for review as listed in Table 1.

### 2.3. Statistics

Statistical analysis was performed using JASP (Version 0.16.3) [16], an open-source statistics program, and BiAS, a commercial statistics program (Version 11.12, © 1989–2021, epsilon-Verlag, Frankfurt, Germany). The Kolmogorov–Smirnov Test [17] was used to determine whether the sample data were normally distributed. Since the sample data were not normally distributed, the Kruskal–Wallis test [17], a rank-based nonparametric test, was used to determine if there were statistically significant differences with regard to age and gender distribution between etiological groups 1–10. The null hypothesis was that there was no difference between etiological groups with regard to age and gender distribution. Since the Kruskal–Wallis null hypothesis was rejected with regard to age distribution, the Dunn’s test for stochastic dominance [18] was used to report the results among multiple pairwise comparisons. *p*-values were adjusted for multiple comparisons using the Holm–Bonferroni method. A *p*-value < 0.05 was considered to be statistically significant.

## 3. Results

### 3.1. Patient Characteristics

Overall, 5166 pediatric patients received brain scans at our hospital within the defined period. A total of 176 patients with WMSAs were enrolled in the study according to predefined criteria. The mean age in the study population was 9.8 years (min 3 days; max 18.9 years). Ninety-nine patients were female (56%) and 77 patients were male (44%). 

### 3.2. General Imaging Characteristics

**Figure 1:**
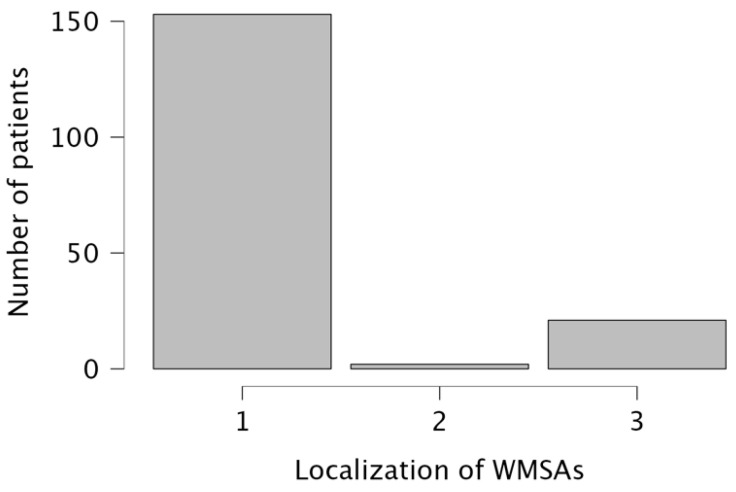
Distribution of WMSAs in the study cohort. 1 = supratentorial only; 2 = infratentorial only; 3 = combination of supra- and infratentorial WMSAs.

WMSAs were divided according to their localization into supratentorial white matter regions only *n* = 153 (87%), infratentorial regions only *n* = 2 (1%), and a combination of supra- and infratentorial regions *n* = 21 (12%). Results are visualized in Figure 1.

In 138 patients, the application of Gadolinium-based contrast agents was indicated (78% of study population). Pathological contrast enhancement on post-contrast T1W images was present in 31 scans (22%).

### 3.3. Etiology-Based Classification of WMSAs

Table 2 shows an etiology-based classification of the WMSAs encountered in the study population.

### 3.4. Age and Gender Distribution of WMSAs

**Figure 2:**
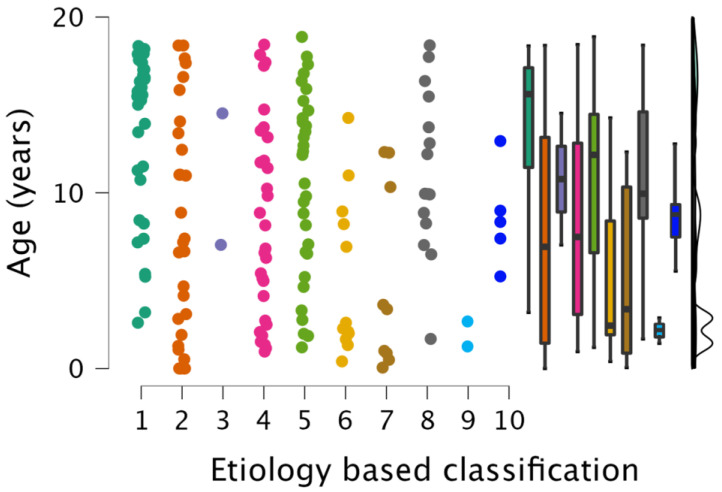
Raincloud plot to visualize the distribution of the data and key summary statistics. The raincloud plot combines a cloud of points with a box plot and a one-sided violin plot.

Etiology-based classification of WMSAs was not affected by gender. It was, however, significantly affected by age (*p* < 0.001). Pairwise comparison showed significant differences between groups 1 (autoimmune disorders) and 2 (non-genetic hypoxic and ischemic insults), 1 and 6 (infectious white matter damage), and 1 and 7 (leukodystrophies). Group 4 was not reported on, as it consisted of patients for whom a definite diagnosis could not be established due to insufficient clinical information. Distribution of the data and key summary statistics are visualized in Figure 2.

### 3.5. Individual Group Characteristics

#### 3.5.1. Autoimmune Disease (Group 1)

**Figure 3:**
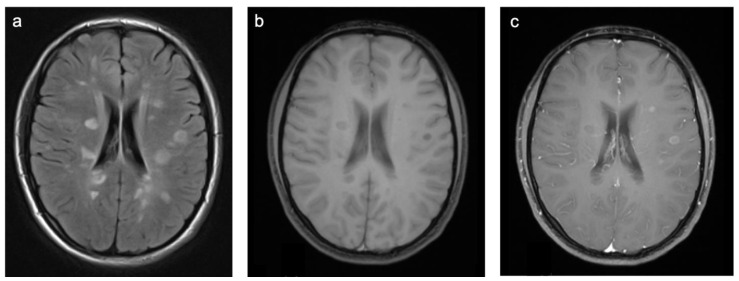
Representative case “Autoimmune disorders”: MS. FLAIR (**a**), T1W pre- and post-contrast (**b**,**c**) sequences. Fifteen-year-old girl diagnosed with MS. MRI shows characteristic ovoid/round lesions with asymmetric distribution abutting the lateral ventricles (periventricular) and touching the cortex (juxtacortical) [19] (**a**). Contrast-enhancing lesions in T1W post-contrast sequences are considered markers of blood–brain barrier breakdown (**c**).

Group 1 consisted of 40 patients. Twenty-five (63%) patients suffered from multiple sclerosis (MS) and nine (23%) from acute demyelinating encephalomyelitis (ADEM). Three patients were diagnosed with a clinically isolated syndrome, one with limbic encephalitis, one with Rasmussen’s encephalitis, and one with vasculitis. Representative cases are shown in Figure 3. A total of 63% of patients were female and the mean age was 14.0 years (min 2.6 years; max 18.4 years). All but one patient were examined with contrast-enhanced MRI and 44% of those examinations showed at least one enhancing lesion. WMSAs were localized supratentorial in 65% of patients and supra- and infratentorial in 35% of patients. There were no isolated infratentorial lesions. In total, 90% of patients had multifocal WMSAs.

#### 3.5.2. Non-Genetic Hypoxic and Ischemic Insults (Group 2)

**Figure 4:**
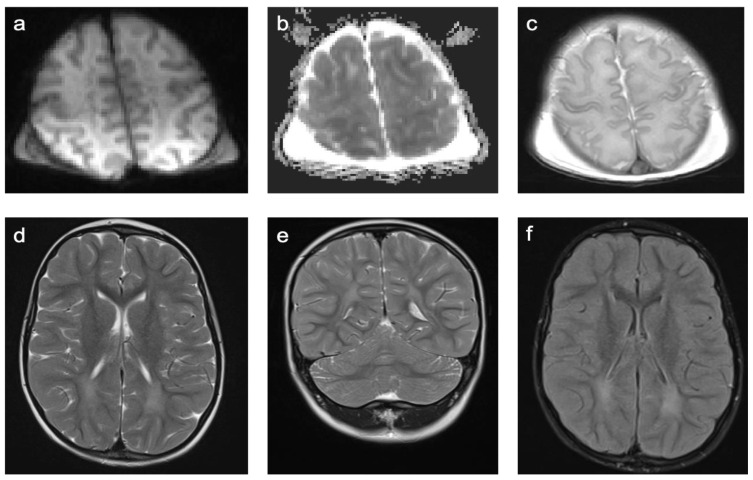
Representative case “Non-genetic hypoxic and ischemic insults”: term-birth asphyxia. DWI/ADC (**a**,**b**), T2W (**c**–**e**), FLAIR (**f**) sequences. Neonatal resuscitation after severe term-birth (40 weeks) asphyxia in a three-day-old girl. (**a**–**c**). Treatment with therapeutic hypothermia. MRI shows predominantly white matter injury as one of the patterns identified in term-birth asphyxia [20]. The same girl examined again at the age of four years (**d**–**f**). MRI shows periventricular signal hyperintensities in T2W/FLAIR sequences.

Group 2 consisted of 30 patients. Eight patients (27%) suffered from perinatal asphyxia; one of them was born preterm. Ten patients (33%) were diagnosed with periventricular leukomalacia (PVL); six of them were born preterm and two had co-occurring intraventricular hemorrhage (IVH). Ten patients (33%) showed signs of ischemic stroke. Eight of these strokes were of obscure or of unknown origin (cryptogenic). One was suspected to be cardio-embolic in a patient with a congenital heart disease with a ventricular septal defect in inherited Xq24 microdeletion syndrome and one as associated with sickle cell disease. The remaining two patients (7%) suffered from a cerebral venous sinus thrombosis with venous outflow obstruction leading to ischemia. A representative case is shown in Figure 4. In total, 60% of patients were female and the mean age was 7.8 years (min 3 days; max 18.4 years). A total of 57% of patients were examined with contrast-enhanced MRI and 12% of those examinations showed at least one enhancing lesion. WMSAs in this group of patients were localized supratentorial only. A total of 43% of patients had multifocal WMSAs.

#### 3.5.3. Traumatic White Matter Injuries (Group 3)

**Figure 5:**
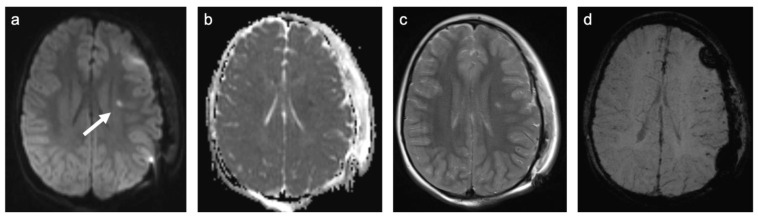
Representative case “Traumatic white matter injuries”: diffuse axonal injury. DWI/ADC (**a**,**b**), T2W (**c**), and Susceptibility weighted imaging (SWI; (**d**)) sequences. Fourteen-year-old girl admitted after a traumatic car accident during which she was ejected from her seat. MRI shows a diffusion restricted, diffuse axonal injury lesion in the lobar white matter ((**a**–**c**), white arrow). SWI demonstrates small regions of susceptibility artefacts at the grey–white matter junction corresponding to cerebral microbleeds (**d**). Additional findings are a subgaleal fluid collection (left side) following craniotomy for treatment of cranial impression fracture.

There were only two patients in Group 3. One patient had a history of traumatic brain injury at the age of 1.5 years, the other patient was admitted after a traumatic car accident. A representative case is shown in Figure 5. Both patients were female. They were 7 and 14.5 years old. One patient was examined with contrast-enhanced MRI. None of the examinations showed enhancing lesions. WMSAs in this group of patients were localized supratentorial only. Both patients had multifocal WMSAs.

#### 3.5.4. Patients with No Final Diagnosis Due to Insufficient Clinical Information (Group 4)

In 30 patients (17% of the study population), a definite diagnosis could not be established due to insufficient clinical information. Group 4 largely consisted of radiology consults to clarify the interpretation of prior imaging studies and diagnoses. In the remaining cases, there was more than one diagnostic possibility at the time of discharge and no follow-up information obtainable. The most common reasons for neuroimaging in this group were non-syndromic intellectual disabilities and developmental delays. Approximately half of these patients showed distribution patterns suggesting various forms of leukodystrophies.

#### 3.5.5. Non-Specific WMSAs (Group 5)

**Figure 6:**
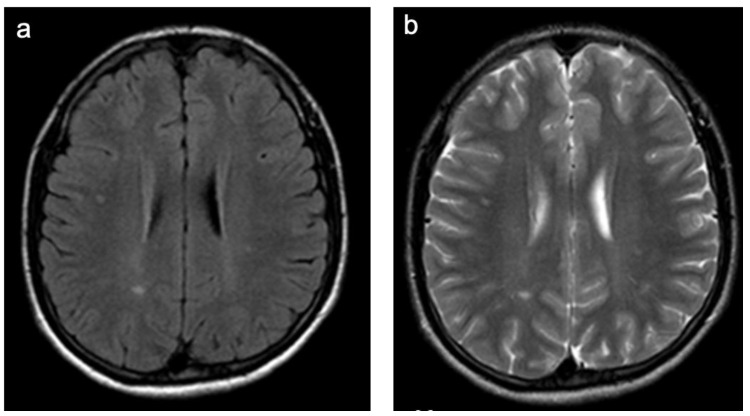
Representative case “Non-specific WMSAs”. FLAIR (**a**) and T2W (**b**) sequences. Fifteen-year-old girl presenting with a headache. MRI shows non-specific periventricular and subcortical WMSAs. Follow-up scan showed stable lesions (**a**,**b**). Patients in this group presented with WMSAs and clinical symptoms that were non-specific for the large number of disorders associated with such abnormalities.

Group 5 consisted of 31 patients. Presenting symptoms that led to the indication of neuroimaging in this group were headaches including migraine attacks (35%), developmental delays (13%, one patient with additional seizures), and seizures (26%). The remaining cases were incidental findings mainly in negative staging MRIs. A representative case is shown in Figure 6. In total, 52% of patients were female and the mean age was 10.5 years (min 1.2 years; max 18.9 years). A total of 65% of patients were examined with contrast-enhanced MRI. None of the examinations showed enhancement of the white matter lesions. WMSAs in this group of patients were localized supratentorial only. A total of 48% of patients had multifocal WMSAs. In total, 39% were followed up with brain MRI after a mean of 13 months (range 16 days—5.5 years from baseline) and all but one of them had a second follow-up scan after a mean of 26 months (range 4 months—8.2 years from baseline). All but one follow-up scan showed stable lesions. In this one patient, white matter signal changes were slightly progressive over several years but remained without clinical significance. Therefore, no diagnosis was established.

#### 3.5.6. Infectious White Matter Damage (Group 6)

**Figure 7:**
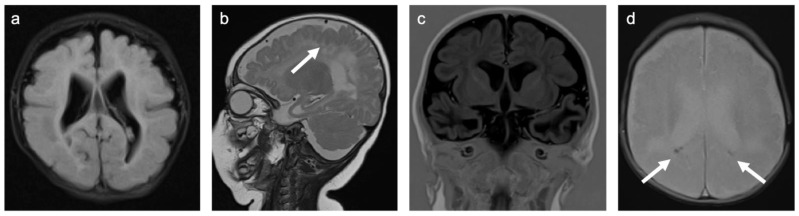
Representative case “Infectious white matter damage”: congenital cytomegalovirus infection. FLAIR (**a**), T2W (**b**), Turbo inversion recovery (TIR; (**c**)), and T2*W (**d**) sequences. Four-month-old girl with a history of congenital cytomegalovirus infection. MRI shows microcephaly with polymicrogyria ((**b**); white arrow), bilateral ventriculomegaly (**a**–**d**), global thinning of the cerebral mantle, especially temporal (**b**,**c**), bilateral white matter signal abnormalities in the frontal and parieto-occipital regions (**a**), and punctuate periventricular calcifications ((**d**); white arrows).

Group 6 consisted of twelve patients. Five patients were diagnosed with congenital cytomegalovirus infections, five with central nervous system manifestations of mycoplasma infection, and two with a central nervous system manifestation of a TORCH infection. A representative case is shown in Figure 7. In total, 33% of patients were female and the mean age was 5.1 years (min 0.4 years; max 14.2 years). A total of 83% of patients were examined with contrast-enhanced MRI. Of those examinations, 20% showed enhancing lesions. WMSAs in this group of patients were localized supratentorial in all but one patient who showed additional infratentorial lesions. A total of 83% of patients had multifocal WMSAs.

#### 3.5.7. Leukodystrophies (Group 7)

Group 7 consisted of nine patients. One patient was diagnosed with a chromosomal duplication of 14q, one with a fatty acid hydrolase-associated neurodegeneration, one with Canavan disease, one with a megalencephalic leukoencephalopathy with subcortical cysts, one with globoid cell leukodystrophy (Krabbe disease), one with 4H leukodystrophy, one with a metachromatic leukodystrophy, and two with x-linked adrenoleukodystrophy. Representative cases are shown in Supplementary Table S1. In total, 44% of patients were female and the mean age was 4.9 years (min 23 days; max 12.3 years). A total of 89% of patients were examined with contrast-enhanced MRI. None of those examinations showed enhancing lesions. WMSAs were localized supratentorial in 67% of patients and supra- and infratentorial in 33% of patients. There were no isolated infratentorial lesions. In total, 89% of patients had multifocal WMSAs.

#### 3.5.8. Toxic White Matter Injuries (Group 8)

**Figure 8:**
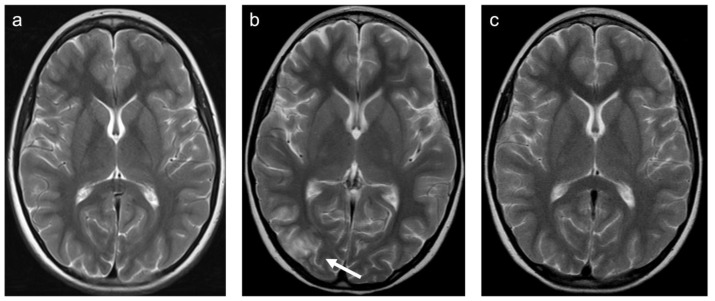
Representative case “Toxic white matter injuries”: L-asparaginase treatment. T2W (**a**–**c**) sequences. ten-year-old boy with induction of L-asparaginase treatment of acute lymphoblastic lymphoblastic leukemia. MRI prior to treatment (**a**), shortly after induction of treatment showing vasogenic edema within the right (white arrow) and, of lesser extent, left occipital region ((**b**); clinical presentation: epileptic seizure) with patchy enhancement in a leptomeningeal pattern (not shown) and restitution after treatment stop (**c**).

Group 8 consisted of 15 patients. Six patients were diagnosed with a posterior reversible encephalopathy syndrome (PRES). Eight were diagnosed with a toxic leukoencephalopathy, predominantly related to high-dose, multidrug chemotherapy. Two cases were related to cranial radiation and immunosuppressive drugs (Ciclosporin A), respectively. A representative case is shown in Figure 8. A total of 60% patients were female and the mean age was 4.6 years (min 1.7 years; max 18.4 years). In total, 93% of patients were examined with contrast-enhanced MRI. Of those examinations, 29% showed enhancing lesions. WMSAs in this group of patients were localized supratentorial only. A total of 87% of patients had multifocal WMSAs.

#### 3.5.9. Inborn Errors of Metabolism (Group 9)

**Figure 9:**
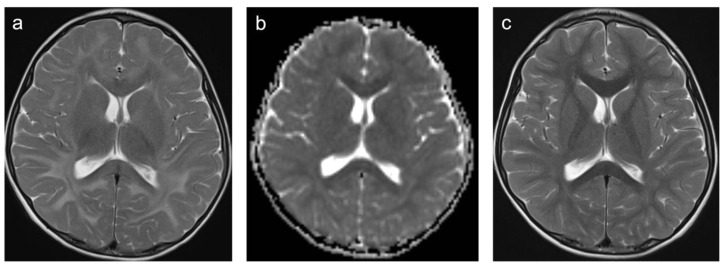
Representative case “Inborn errors of metabolism”: methionine adenosyltransferase I/III deficiency. T2W (**a**,**c**) sequences and ADC map (**b**). Three-year-old boy diagnosed with a methionine adenosyltransferase I/III deficiency. MRI shows T2W signal hyperintensities in subcortical and deep white matter with relative sparing of corticospinal tracts, corpus callosum and optic radiations (**a**). ADC map demonstrates decreased ADC value of the lesion (**b**). Normalization of established WMSAs within two years on a strict methionine-restricted diet (**c**).

There were only two patients in Group 9. One patient was diagnosed with an isovaleric acidemia and one with a methionine adenosyltransferase I/III deficiency. A representative case is shown in Figure 9. In total, 50% of patients were female and the mean age was 2.2 years (min 1.4 years; max 2.9 years). One patient was examined with contrast-enhanced MRI. None of the examinations showed enhancing lesions. WMSAs in this group of patients were localized supratentorial only. All patients had multifocal WMSAs.

#### 3.5.10. White Matter Damage Caused by Tumor Infiltration/Cancer-like Disease (Group 10)

**Figure 10:**
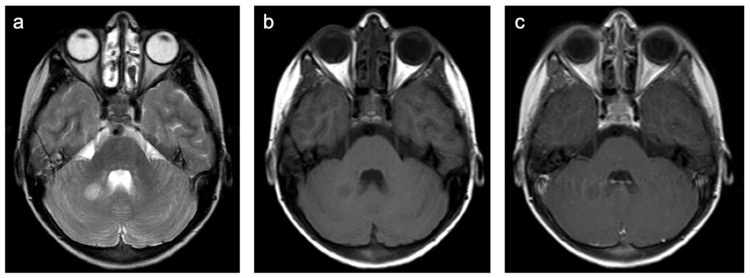
Representative case “White matter damage caused by tumor infiltration”: pediatric type diffuse low-grade glioma. T2W (**a**), T1W pre- (**b**) and post-contrast (**c**) sequences. Ten-year-old girl diagnosed with a pediatric type diffuse low-grade glioma. T2W sequence shows mass-like hyperintense lesion, predominantly located in the white matter of the right cerebellar hemisphere (**a**). No contrast enhancement present (**c**).

Group 10 consisted of five patients. One patient was diagnosed with a pediatric type diffuse low-grade glioma, one with a secondary CNS lymphoma, and one with a neurodegenerative Langerhans cell histiocytosis. In two patients, a low-grade epilepsy-associated tumor (LEAT) was suspected. A representative case is shown in Figure 10. In total, 60% of patients were female and the mean age was 8.8 years (min 5.6 years; max 12.9 years). All patients were examined with contrast-enhanced MRI. None of the examinations showed enhancing lesions. WMSAs in this group of patients were localized supratentorial in 60% of patients and infratentorial in 40% of patients.

## 4. Discussion

In this study, we retrospectively evaluated WMSAs in an unselected patient cohort at a large pediatric neuroimaging facility with regard to imaging characteristics, etiology, as well as age and gender distribution.

Overall, WMSAs were found in 3.4% of pediatric patients scanned at our and referring hospitals within the ten-year study period. The majority of WMSAs were found in the supratentorial region only. Among patients examined with Gadolinium-based contrast agents (approximately 2/3 of the study population), only one out of five patients showed pathological enhancement. WMSAs caused by autoimmune disorders formed the largest group, followed by non-genetic hypoxic and ischemic insults, as well as non-specific WMSAs. The majority of WMSAs was therefore acquired as opposed to inherited. Etiology-based classification of WMSAs was affected by age. Pairwise comparison showed significant age differences between patients with autoimmune disorders and those with non-genetic hypoxic and ischemic insults, infectious white matter damage, and leukodystrophies. These findings were driven by the predominance of adolescent-onset MS patients in the autoimmune group [21,22,23], and the predominance of perinatal insults, congenital infections, and leukodystrophies with early symptom-onset [24] in the other groups. There were no gender related differences. 

While the literature provides numerous reports characterizing WMSAs in children with certain conditions or diseases [11,25,26,27,28,29,30,31], as well as in children with the same group of symptoms [32,33,34], there is little information on WMSAs in an unselected pediatric patient cohort, which limits the contextualization of our results. Kristjánsdóttier et al. provided first insights into the spectrum of WMSAs in children in a Swedish multicenter study in 1996 [35]. In their initial report, they evaluated 78 consecutive children with WMSAs. Approximately 60% of the children did not receive a final diagnosis due to insufficient clinical information, which again stresses the need for an “integrated diagnosis” that combines baseline demographics, clinical characteristics, and additional diagnostic workup with imaging patterns. In their cohort, patients with errors of metabolism formed the largest group with an actual diagnosis, followed by patients with autoimmune disorders. They did, however, identify their patients by an apparent “leukodystrophic” pattern rather than considering all WMSAs, which might explain the discrepancies to our cohort. Another factor could be the limited availability of pediatric MRI in the early 1990s with restricted use. 

Approximately one out of five study patients in our cohort presented with WMSAs that were not considered causative for the reasons of referral, foremost headaches including migraine attacks, developmental delays, and seizures. Overall, this group represented less than 1% of pediatric patient scans at our institution. Our prevalence is therefore lower than the prevalence Dangouloff-Ros et al. found in a large meta-analysis on incidental findings in pediatric MRI studies. They reported non-specific WMSAs in 2% of brain scans [15]. One important take-away that they discuss for all incidental findings holds true for non-specific WMSAs as well: image resolution (signal to noise ratio, spatial resolution) seems to play an important role with regard to reported rates. In addition, layer gaps in 2D as opposed to 3D images can result in smaller lesions being missed [5]. Whether or not these supposably “non-specific” findings could still be related to certain pathologies remains a matter of debate. Reported prevalence in pediatric patients with headaches/migraine attacks seem higher (4–7%) [33,34], but with no statistically significant difference compared to a control group within the same study (7 vs. 4%) [33]. In adult patients (even younger adults), a two- to fourfold increased incidence is commonly accepted, yet pathophysiology of these migraine-related WMSAs remains poorly understood. Both ischemic and inflammatory mechanisms have been proposed [36]. Kjos et al. investigated WMSAs in pediatric patients with intellectual disabilities of unknown cause (5% prevalence) and interpreted them as an indicator of previous, non-specific cerebral insults, but not as the direct cause of the developmental delays [37]. Kalnin et al. described WMSAs in 3% of pediatric patients with a first ever seizure, but considered none of them causative [38].

### Limitations of the Study

Our study is limited by the initial identification of eligible patients: since they were identified by filtering the RIS for exam type and predefined keywords in the respective radiology reports, patients with WMSAs that were not described in the reports were not included. 

Due to the heterogeneity of the data in this retrospective cohort (hardware, protocols) and the—in the context of machine learning/deep learning—relatively small sample size with WMSAs, we decided against these otherwise very informative approaches. In the recent literature they have been primarily used on segmentation tasks for WMSA [39,40,41], but some work seems to emerge on classification tasks as well [42].

Lastly, inborn errors of metabolism might be underrepresented, as the University Hospital Frankfurt is not a pediatric metabolic disease specialty center.

## 5. Conclusions

Over the past two decades, the rapidly expanding use of MRI in children with neurological impairments of unknown etiology has revealed a substantial number of WMSAs. An “integrated diagnosis” that combines baseline demographics, including patient age as an important factor, clinical characteristics and additional diagnostic workup with imaging patterns can be made in the majority of cases. Unselected patient cohorts are dominated by acquired, as opposed to inherited, white matter lesions. WMSAs that are not interpreted as the direct cause of the presenting symptoms are often categorized as “non-specific”, even though they may still be related to certain pathologies (i.e., ischemic and inflammatory mechanisms in patients with migraines).

## Figures and Tables

**Table 1:** Group of basic MRI sequences available for review. T1W = T1-weighted; T2W = T2-weighted; SE = spin echo; FSE = fast spin echo; TSE = turbo spin echo; TIRM = turbo inversion recovery magnitude; df = dark fluid; FLAIR = fluid-attenuated inversion recovery; DWI = diffusion-weighted imaging; ADC = apparent diffusion coefficient.

Sequence	Plane	Comment
2D T1W SE or FSE (3D MPRAGE in a subset of patients)	axial	pre- and, if clinically indicated, post-contrast (Gadolinium-based contrast agents)
2D T2W TSE	axial	
FLAIR or TIRM df	axial	
DWI/ADC	axial	b-values 0, 1000

**Table 2:** Etiology-based classification of WMSAs encountered in the study population. Number of patients and percentage of study population. Percentage rounded to the nearest whole number.

Etiology of WMSAs	Number of Study Patients (*n*)	Percentage of Study Population
Autoimmune disease (Group 1)	40	23%
Non-genetic hypoxic and ischemic insults (Group 2)	30	17%
Traumatic white matter injuries (Group 3)	2	1%
Patients with no final diagnosis due to insufficient clinical information (Group 4)	30	17%
Non-specific WMSAs (Group 5)	31	18%
Infectious and post-infectious white matter damage (Group 6)	12	7%
Leukodystrophies (Group 7)	9	5%
Toxic white matter injuries (Group 8)	15	9%
Inborn errors of metabolism (Group 9)	2	1%
White matter damage caused by tumor infiltration/cancer-like disease (Group 10)	5	3%

## Data Availability

Authors agree to make data and materials supporting the results or analyses presented in their paper available upon reasonable request.

## Supplementary Materials

The following supporting information can be downloaded at: https://www.mdpi.com/article/10.3390/children10020206/s1, Supplementary Table S1: Representative examples of heritable disorders affecting the white matter of the central nervous system, defined as leukodystrophies (Group 7). T2W sequences.

## Abbreviation

ADEM	acute demyelinating encephalomyelitis
ADC	apparent diffusion coefficient
APS	antiphospholipid syndrome
CIS	clinical information system
DNET	dysembryoplastic neuroepithelial tumor
DTI	diffusion tensor imaging
DWI	diffusion-weighted imaging
WMSA	white matter signal abnormality
FA	fractional anisotropy
FLAIR	fluid-attenuated inversion recovery
FSE	fast spin echo
IRB	institutional review board
IVH	intraventricular hemorrhage
MS	multiple sclerosis
PRES	posterior reversible encephalopathy syndrome
SE	spin echo
RIS	radiological information system
T	Tesla
TIRM	turbo-inversion recovery-magnitude dark fluid
TSE	turbo spin echo
T1W	T1-weighted
T2W	T2-weighted
