# Supplementary material for: Characterization of MRI White Matter Signal Abnormalities in the Pediatric Population
Source: Children (Basel). 2023 Jan 24;10(2):206. doi: 10.3390/children10020206 (PMC9955075; doi:10.3390/children10020206)
Supplement: Supplementary file 1 [file children-10-00206-s001.zip › children-2044514-supplementary.pdf]

Supplement

Genetic white matter disorder	Imaging findings
Canavan disease (aspartoacylase deficiency)<br><br>Leukodystrophy with myelin vacuolization	Diffuse, bilateral WMSAs involving the subcortical U-fibers, sparing the corpus callosum, caudate nucleus, putamen and internal capsule<br>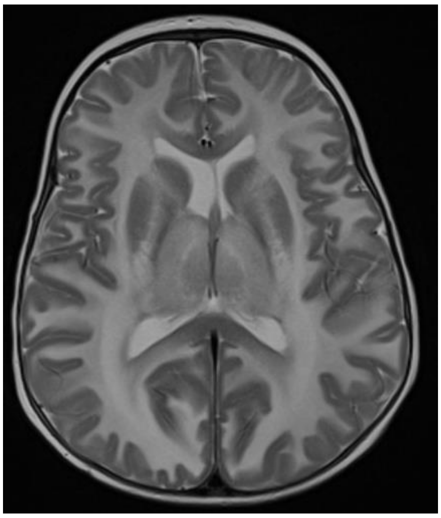<br>6-months-old boy
4H leukodystrophy<br><br>Hypomyelination	Diffuse bilateral WMSAs. Relative T2 hypointensity of the ventrolateral thalamus (white arrows) and of the optic radiation (white striped arrow); thinning of the corpus callosum but no cerebellar atrophy (yet)<br>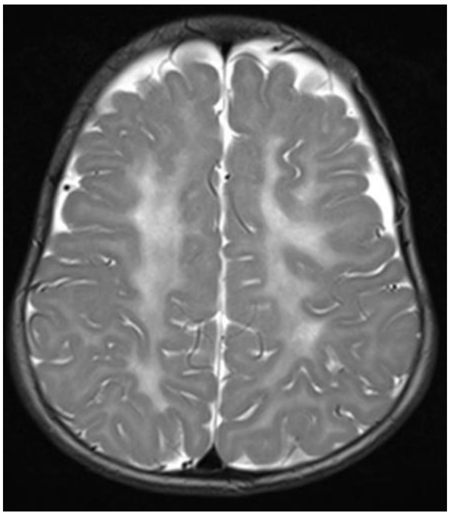

	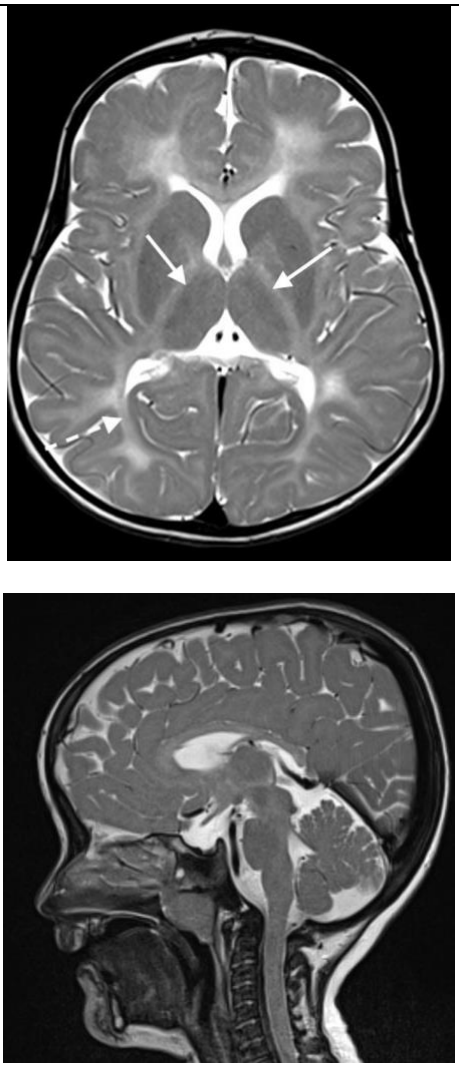<br>3.6-year-old girl

Globoid cell leukodystrophy (Krabbe disease)<br><br>Hypomyelination	Relative T2 hyperintensity involving the periventricular white matter, centrum semiovale and deep grey matter; subcortical U-fibers at this early stage spared<br>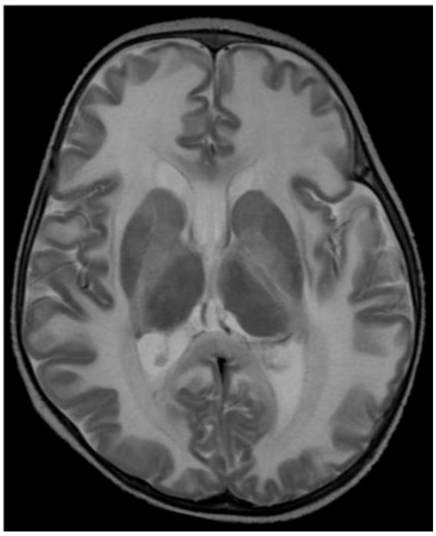<br>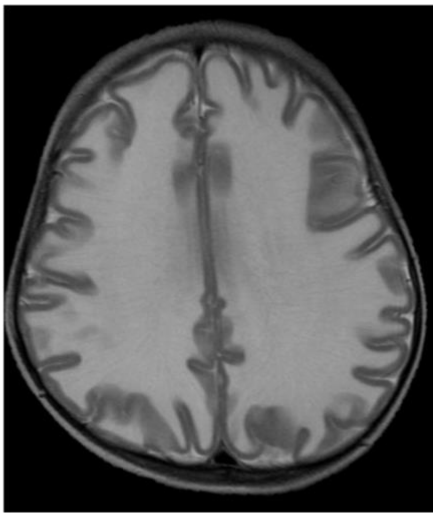<br>12-months-old boy

X-linked adrenoleukodystrophy (metabolic peroxisomal disorder)<br><br>Hypomyelination	Deep WMSAs in the parieto-occipital lobes and splenium of the corpus callosum (most common pattern); in the craniocaudal direction signal abnormality is seen in the corticospinal tracts extending to the level of the pyramids of the medulla<br>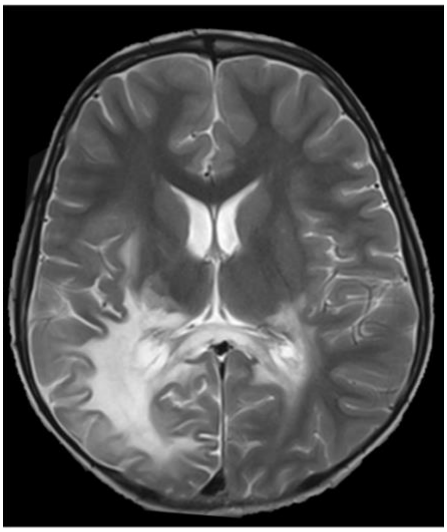<br>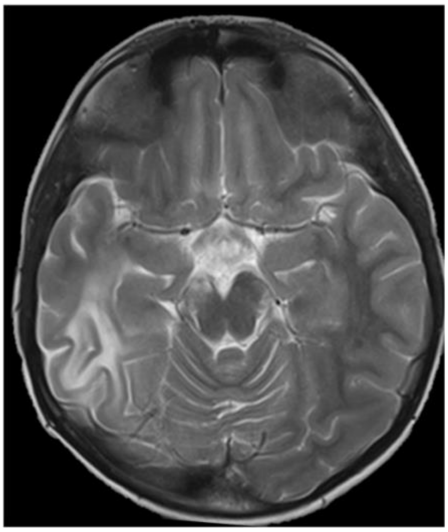<br>10.3-year-old boy

Metachromatic leukodystrophy<br><br><br>Hypomyelination	Bilateral symmetrical confluent deep WMSAs, in particular around the atria and frontal horns with sparing of subcortical U-fibers leading to a "butterfly pattern"<br>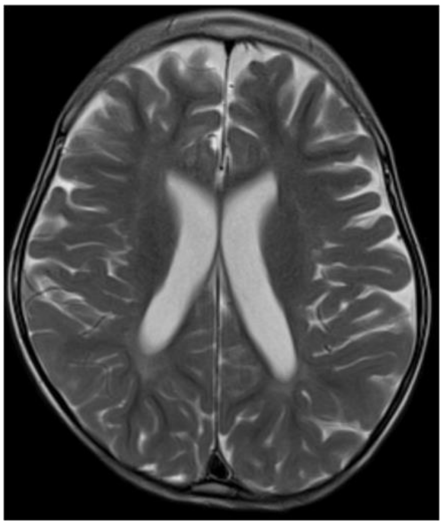<br>3.4-year-old girl

Supplementary Table 1. Representative examples of heritable disorders affecting the white matter of the central nervous system, defined as leukodystrophies (Group 7). T2W sequences.
